# Sleep Disordered Breathing challenges: From diagnosis to treatment

**DOI:** 10.5935/1984-0063.20180024

**Published:** 2018

**Authors:** Geraldo Lorenzi Filho, Pedro Rodrigues Genta

**Affiliations:** 1 Sleep Laboratory, Pulmonary Division, Heart Institute (InCor), Hospital das Clinicas HCFMUSP, Faculdade de Medicina, Universidade de São Paulo, São Paulo, SP, BR.

This issue of Sleep Science brings three Latin American articles that deal with contemporary challenges in the diagnosis and management of sleep disordered breathing (SDB). In one of the studies, a simple approach to facilitate OSA diagnosis and shortcut the initiation of CPAP treatment is tested^1^. In another study, overall adherence to CPAP is explored, focusing on the prevalence and motivations of failure to initiate CPAP after prescription^2^. The third study describes sleep disordered breathing among patients with decompensated heart failure in Bogota, a city located at the Andes at 2640m above sea level^3^.

It is now evident that obstructive sleep apnea (OSA) is much more common in the general population than previously imagined, ranging from 17% (apnea-hypopnea index[AHI]>15 events/h) to around 40% (AHI>5 events/h)^4^. More than just a common disease, treatment with CPAP abolishes OSA, improves symptoms and may also impact positively on long term cardiovascular consequences. However, the vast majority of patients remains undiagnosed. Reasons for underdiagnosis include insufficient clinical suspicion and impaired access to OSA diagnosis. OSA diagnostic methods ranges from full polysomnography, portable respiratory monitoring and overnight oximetry. In this issue of Sleep Science, Borsini and collaborators propose that simple questionnaires (STOP-BANG) and Epworth Sleep Scale (ESS) made it possible to indicate CPAP reliably (low rate of false-positive results) in 20-40% of patients referred for suspected OSA without a diagnostic test^1^. This information is of relevance in clinical practice because the queues for in-lab polysomnography are long. A simpler and more affordable approach for OSA management is highly wanted and may eventually improve the gap between CPAP prescription and CPAP use initiation.

In another study of the current issue of Sleep Science, Nogueira and collaborators show that most compliance studies often only include patients under CPAP treatment, neglecting the importance of access to treatment^2^. The authors show that after in-lab polysomnography (PSG) and CPAP titration in a private sleep center in Buenos Aires, 28% did not start using CPAP. Possible explanations were extracted from the differences between those who started and those who did not start CPAP and included less severe sleepiness, lack of insurance coverage and higher therapeutic pressures. Among those starting CPAP, 78% were using it after one year^2^. The reported adherence to CPAP (including only those who started therapy) is higher than many previous reports^5^. Improving access to CPAP device is fundamental. It is our duty to convince insurance providers and the government that CPAP is cost-effective and reduces health-associated costs among OSA patients.

While OSA is by far the most common sleep disordered breathing condition, patients with congestive heart failure (CHF) frequently present central sleep apnea^6^. Low PaCO_2_ plays a critical role in the genesis of central sleep apnea among patients with CHF^7^. Interestingly enough, high altitude decreases PaO_2_, induces hyperventilation and lower PaCO_2_. Cities sitting at high altitudes such as Bogota, are the ideal place to ask a simple question: What happens to breathing during sleep when the 2 conditions (CHF and high altitude) are present? In this issue of Sleep Science, Vargas-Ramirez and collaborators studied 16 patients hospitalized for decompensated heart failure in Bogota^3^. The main finding of this study is that all patients included had sleep apnea. Most had severe sleep apnea (75%). Fifty-percent had central sleep apneas and 44% had Cheyne-Stokes respiration that was frequently associated with severe oxygen desaturation. Future studies, comparing patients at low and high altitude, would be an interesting next step.

It is important to highlight that, more than a “local” problem, the articles published in this issue of Sleep Science deals with contemporary questions that are of global concern.
